# Detection of Oculomotor Dysmetria From Mobile Phone Video of the Horizontal Saccades Task Using Signal Processing and Machine Learning Approaches

**DOI:** 10.1109/access.2022.3156964

**Published:** 2022-03-04

**Authors:** HAMED AZAMI, ZHUOQING CHANG, STEVEN E. ARNOLD, GUILLERMO SAPIRO, ANOOPUM S. GUPTA

**Affiliations:** 1 Department of Neurology, Massachusetts General Hospital, Harvard Medical School, Boston, MA 02129, USA; 2 Department of Electrical and Computer Engineering, Duke University, Durham, NC 27707, USA; 3 Department of Computer Science, Duke University, Durham, NC 27707, USA; 4 Department of Biomedical Engineering, Duke University, Durham, NC 27707, USA; 5 Department of Mathematics, Duke University, Durham, NC 27707, USA; 6 Department of Neurology, Massachusetts General Hospital, Harvard Medical School, Boston, MA 02114, USA

**Keywords:** Mobile phone video, horizontal saccades, eye movement analysis, entropy, functional connectivity, template matching, test-retest reliability

## Abstract

Eye movement assessments have the potential to help in diagnosis and tracking of neurological disorders. Cerebellar ataxias cause profound and characteristic abnormalities in smooth pursuit, saccades, and fixation. Oculomotor dysmetria (i.e., hypermetric and hypometric saccades) is a common finding in individuals with cerebellar ataxia. In this study, we evaluated a scalable approach for detecting and quantifying oculomotor dysmetria. Eye movement data were extracted from iPhone video recordings of the horizontal saccade task (a standard clinical task in ataxia) and combined with signal processing and machine learning approaches to quantify saccade abnormalities. Entropy-based measures of eye movements during saccades were significantly different in 72 individuals with ataxia with dysmetria compared with 80 ataxia and Parkinson’s participants without dysmetria. A template matching-based analysis demonstrated that saccadic eye movements in patients without dysmetria were more similar to the ideal template of saccades. A support vector machine was then used to train and test the ability of multiple signal processing features in combination to distinguish individuals with and without oculomotor dysmetria. The model achieved 78% accuracy (sensitivity= 80% and specificity= 76%). These results show that the combination of signal processing and machine learning approaches applied to iPhone video of saccades, allow for extraction of information pertaining to oculomotor dysmetria in ataxia. Overall, this inexpensive and scalable approach for capturing important oculomotor information may be a useful component of a screening tool for ataxia and could allow frequent at-home assessments of oculomotor function in natural history studies and clinical trials.

## I. INTRODUCTION

Neurodegenerative diseases affect oculomotor function in a variety of ways, which impact vision and also provide clues into the underlying pathology and diagnosis [1]–[4]. Eye movements, specifically saccades, can provide insight into both motor control and cognitive function [5]. Eye movement behavior has been studied in a number of neurodegenerative diseases, including Alzheimer’s disease, Parkinson’s disease, multiple sclerosis, Huntington’s disease, and cerebellar ataxia [6]–[13], demonstrating utility for diagnosis and monitoring of disease.

Cerebellar ataxias are a heterogeneous group of inherited and acquired neurologic diseases. Genetic ataxias include autosomal recessive conditions such as ataxia-telangiectasia and Friedreich’s ataxia and autosomal dominant conditions, most notably the spinocerebellar ataxias. As a broad group, ataxias cause profound and characteristic abnormalities in smooth pursuit (slow eye movements that enable following of a moving object), fixation (steadily holding gaze on a stationary target), and saccades [11], [14], [15]. Saccades are characterized by quick eye movements that shift gaze from one fixation point to another and play an important role in attention and information processing tasks such as reading and scene perception [16].

Dysmetria - lack of coordination of movement typified by undershoots or overshoots of the intended position with arms, legs, or eyes - is a hallmark feature of cerebellar ataxia [17]–[19]. Hypermetric/overshoot and hypometric/undershoot saccadic eye movements are prevalent in cerebellar ataxias [15]. Given that individuals with ataxia may have hypometric, hypermetric, or hypometric and hypermetric saccades, these two types of saccadic abnormalities are grouped together in clinical ataxia rating scales [20], [21], and we refer to them collectively as “oculomotor dysmetria”. Although oculomotor dysmetria, especially overshoot (i.e., hypermetric saccades), can probably occur with lesions elsewhere in the brain and in acute injury (e.g., concussion), they are relatively specific for cerebellar pathology [22], [23]. In healthy individuals, saccades slightly undershoot a fixation target (i.e., they are hypometric) whereas hypermetric saccades very rarely occur [24]. In hypermetric saccades, the initial saccade amplitude is too large and dynamically overshoots the target before returning to the target. In hypometric saccades, the initial saccade amplitude is too small leading to production of a corrective saccade in the same direction in order to reach the target. Although very slight undershoot can be present in healthy individuals, the degree of undershoot is often pronounced in individuals with ataxia, such that it can be observed by the clinician during the bedside examination. These pathologic undershoot saccades are what we refer to as hypometric saccades. In a study of 47 patients with cerebellar ataxia, saccadic eye movement dysmetria (i.e., hypometric and/or hypermetric saccades) was present in 76.6% of individuals [25] and other studies have shown that dysmetric saccades are prevalent features in spinocerebellar ataxias [15], [26].

Given the prevalence of oculomotor abnormalities in ataxia, quantitative assessments of saccades could be useful in screening for ataxia and for assessing severity of disease state over time. However, the need for specialized and expensive eye tracking equipment as well as expertise in operating the devices, prevents widespread use of eye tracking. With these constraints, clinical studies of eye movement disturbances in neurologic diseases usually include small numbers of participants at a single time point. Eye tracking using consumer-grade electronic devices provides an opportunity to increase accessibility and reduce burden of quantitative oculomotor assessments for both screening and severity monitoring purposes, and may allow for monitoring in the home setting [27], [28]. In this study, we tested whether signal processing methods based on template matching, information theory (complexity- and irregularity-based techniques), and functional connectivity, applied to eye tracking data collected from a mobile phone camera could enable detection of oculomotor dysmetria.

## II. METHODS

In this Section, the materials and methods used in this study are briefly described.

### A. MATERIALS

We collected video data on 175 ataxia and Parkinson’s participants recorded in eye movement testing in Massachusetts General Hospital (MGH) Neurology clinics. Individuals were evaluated by a neurologist on the day of the video data collection and information about the presence or absence of hypo/hypermetric saccades was extracted from the electronic medical record. In rare cases that saccades were not evaluated, the video recordings of saccades were evaluated by a trained neurologist. If an individual had pathologic hypometric or hypermetric saccades, they were labeled as having dysmetric saccades. We excluded the data from 23 participants whose data was not at least 42 s or who did not follow the task. Of the 152 participants whose data we used for the analysis, 72 (38 females) had cerebellar ataxia with dysmetria, 37 (14 females) had ataxia without dysmetria and 43 (12 females) had Parkinson’s without dysmetria. The mean ± standard deviation age of the subjects with and without dysmetria were 44.75 ± 21.80 and 63.19 ± 15.15, respectively. The number of sessions was 2 for 12 individuals and 1 for 140 subjects.

Stimuli for the horizontal saccades task were presented on an Apple iPad screen, while simultaneously recording each participant’s face from an Apple iPhone camera sampling at 240fps [29]. Participants were seated approximately one foot in front of the 12.9-inch screen. The task included 20 trials of horizontal saccades, with the target beginning at the center of the screen, moving to the left or right of the screen (16 degree amplitude), and then returning to the screen center. The order of left and right trials was pseudorandom and the target duration was uniformly distributed between 1.3–2 seconds for each presentation. The video was processed using [30] to detect 12 eye and 2 iris center facial landmarks for each frame in an individual’s video [29]. To account for head movement, we computed, for each eye, the normalized iris center (NIC) as the iris center position relative to the midpoint of the 2 eye corner landmarks. We use the X (horizontal) coordinate of the NIC corresponding to frames when the dot moved horizontally in subsequent processing steps due to it having much higher signal to noise ratio compared to the Y (vertical) coordinate. For more information, please see [29]. The length of the data is 42s for each subject. As in vertical data, eyelid close, particularly with downgaze, sometimes blocks eye tracking or at least decreases the quality of iris tracking, we analyzed only the horizontal eye movement.

**FIGURE 1.**
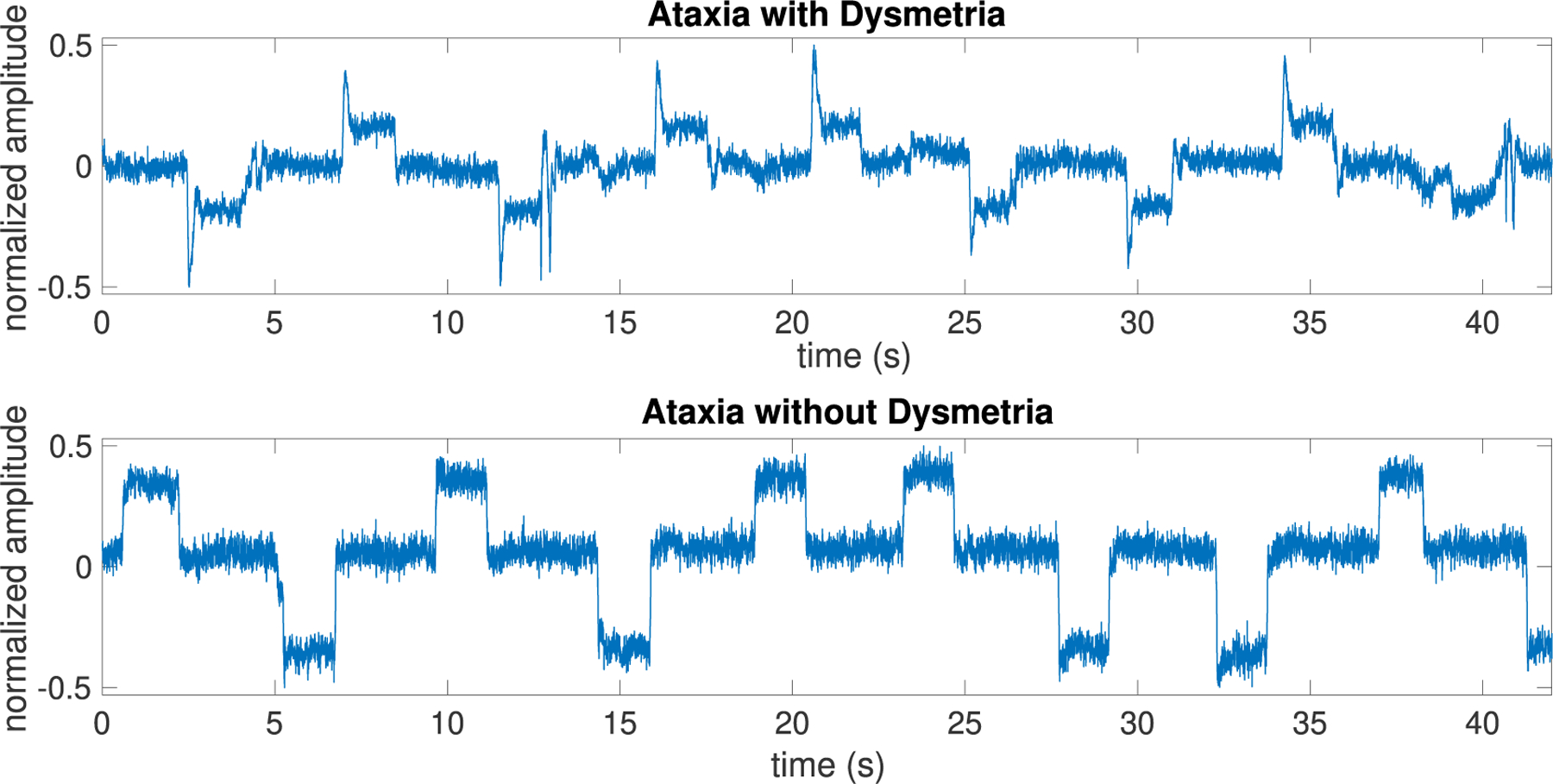
Example of saccadic eye movement after normalizing to the range [−0.5, +0.5] for an ataxia patient with dysmetria vs. an ataxia subject without dysmetria.

Visual inspection of eye tracking data extracted from iPhone video recordings of ataxia and Parkinson’s participants provided evidence that the technology could capture hypermetric and hypometric saccades. An example of saccadic eye movement for an ataxia participant with hypermetric saccades vs. an ataxia participant without dysmetric saccades is depicted in Figure 1. To have a data within a specified range, we first scaled the data to fall in the range [−0.5,+0.5]. In this way, we have more reliable results as template matching and some functional connectivity methods are too sensitive to amplitude values. Note that in the detection of oculomotor dysmetria, we need to detect amplitude change rather than amplitude values.

### B. POWER SPECTRAL DENSITY AND PRE-PROCESSING STEP

**FIGURE 2.**
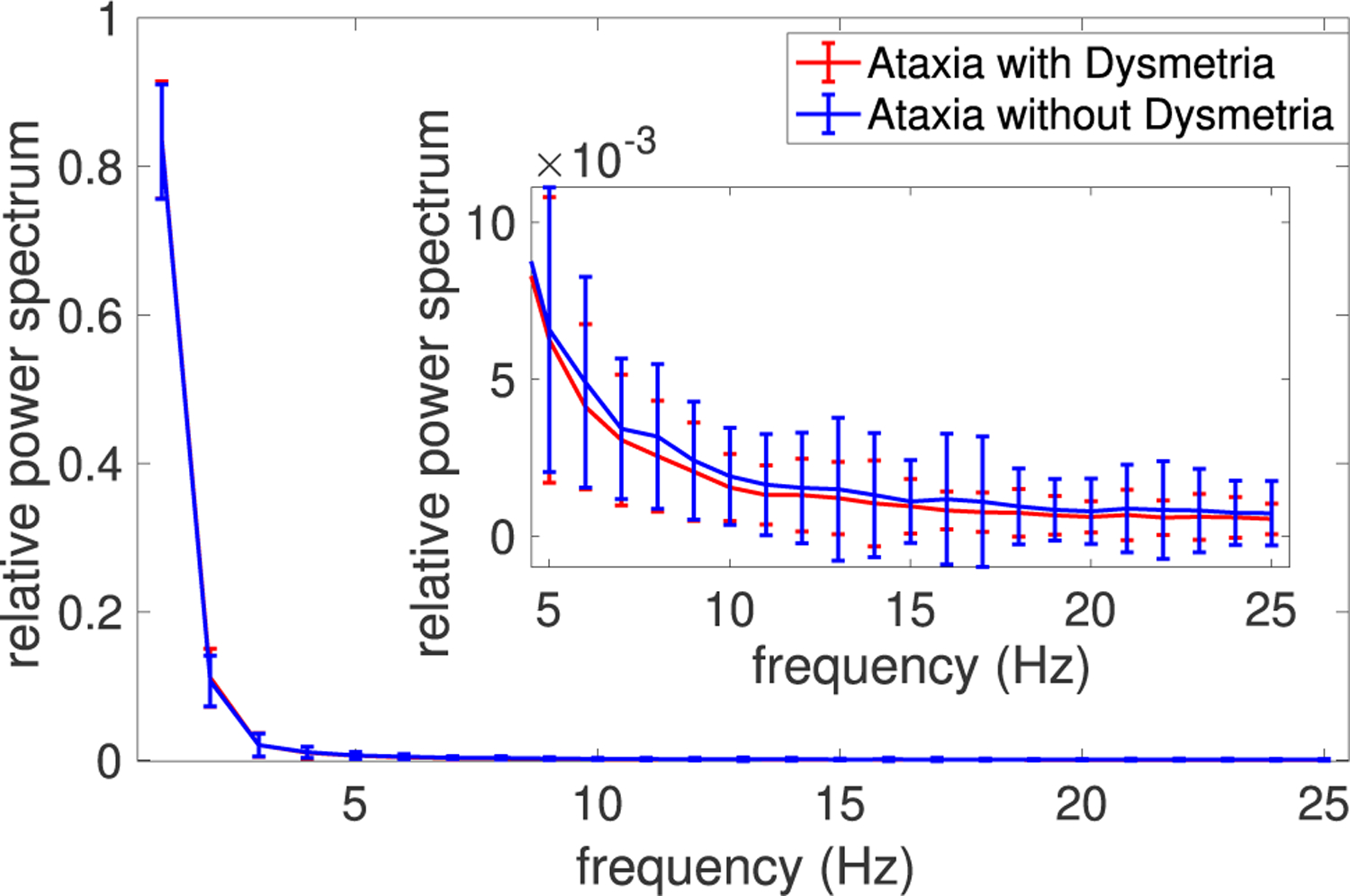
Relative power spectral density for individuals with vs. without dysmetria.

**FIGURE 3.**
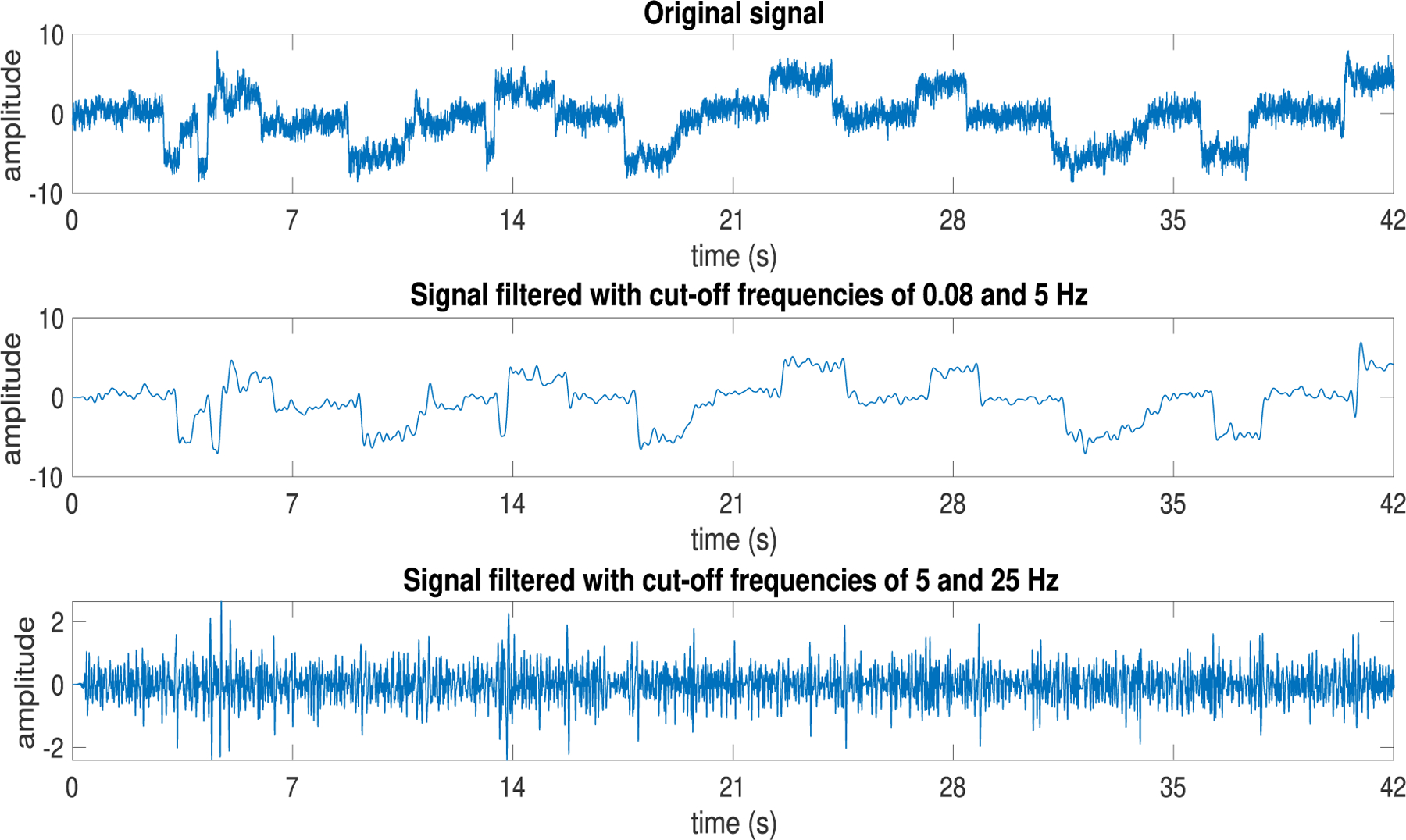
Original eye-movement data, and its filtered forms with cut-off frequencies of 0.08 and 5 Hz, and 5 and 25 Hz.

The relative power spectral density profiles of the eye tracking data for individuals with and without oculomotor dysmetria are shown in Figure 2. The difference between the two groups was not significant using the Mann-Whitney *U* test. Visual inspection of the power spectral densities demonstrated more power in the lower frequency components (1–5 Hz). This is in agreement with observations that although eye movement activity has a wide frequency range, there is increased power at low frequencies (< 5 Hz) [31], [32]. Thus, we evaluated the data in two distinct frequency bands 0.08 – 5 Hz and 5 – 25 Hz [33]. The cut-off frequency of 0.08 Hz was chosen in order to remove low-frequency drifts [12]. An example eye movement tracing showing the original and filtered tracings in the two frequency bands is shown in Figure 3.

Thus, we first used two 200th FIR band-pass filters with a Hamming window and frequency bands 0.08 to 5 Hz and 5 to 25 Hz. We then used an approach based on a median filter with order 200 for the removal of eye blinks. As the median filter removes blinks, overshoots and undershoots, we removed any abnormality that increases and decreases to its initial value (only eye-blinks rather than overshoots or undershoots).

### C. MATCHED FILTERING

**FIGURE 4.**
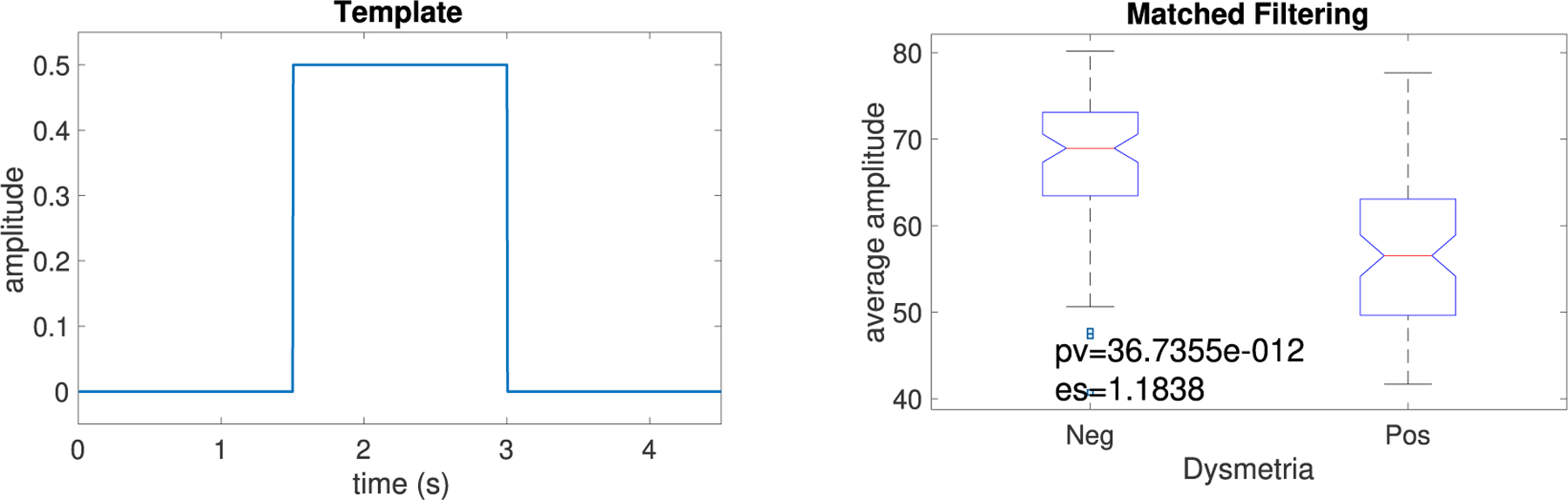
Template used for the matched filter and the boxplot of the average amplitude values for the patients with and without saccadic dysmetria in the frequency band 0.08–5 Hz and their corresponding *p*-value and effect size.

We also use the concept of matched filtering to differentiate between some wanted patterns (Figure 4) and random or unwanted patterns [34]. A matched filter is obtained by correlating a known delayed time series, or template, with an unknown time series to detect the presence of the template in the unknown time series [35], [36]. In this study, the concept of matched filtering was used to compare saccades with the “ideal” template signal shown in Figure 4. In every 42s saccadic eye-movement, there are at least four saccades from center fixation to the left target and back to the center (same for the right target). Therefore, we considered the first four maximum and absolute first four minimum values of the filtered data (see Figure 4).

### D. THRESHOLD-BASED SURFACE

**FIGURE 5.**
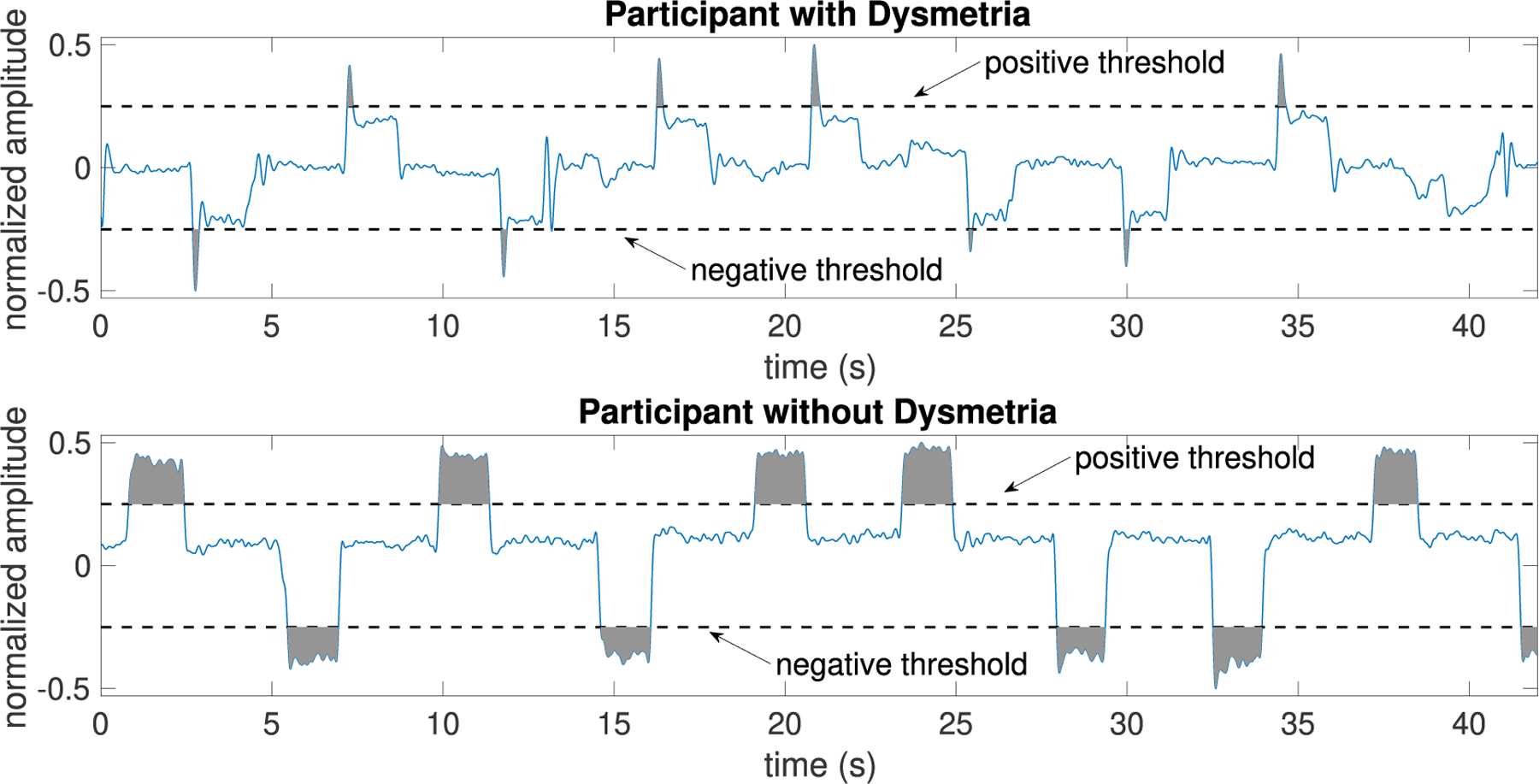
Eye movement data with vs. without dysmetria (Figure 1) band-pass filtered with cutoff frequencies 0.08 and 5Hz. Shaded regions are used to detect saccadic dysmetria.

A threshold-based approach was used to identify and quantify the degree of overshoot present in individuals with hypermetric saccades. Given that hypermetric saccades produce a sharp peaked signal, we predicted that the area between the eye tracking curve and a threshold would be small in individuals with hypermetric saccades compared with individuals without hypermetric saccades. The original eye tracking data were band-pass filtered with the cut-off frequencies 0.08 and 5 Hz (Figure 5). After normalizing the data to the range [−0.5,+0.5], the area above the positive threshold and the signal plus the area below the negative threshold and the signal were calculated (see shaded regions in Figure 5). As expected, the surface area for ataxia participants with oculomotor dysmetria was significantly smaller than that for ataxia participants without dysmetria.

### E. ENTROPY, FRACTAL DIMENSION, AND LEMPEL-ZIV COMPLEXITY

Entropy, fractal dimension (FD), and Lempel-Ziv complexity (LZC) are three general nonlinear signal processing methods which are widely used in biomedical applications to quantify the irregularity or uncertainty of data. LZC is a widespread metric to analyze biological signals to evaluate the complexity or regularity of finite sequences [37]. LZC is associated with the number of distinct substrings and the rate of their recurrence along the analysed time series, with larger values corresponding to more complex or irregular data [37]. FD refers to a noninteger or fractional dimension of a geometric object [38]. In this study, we use [39] as a popular technique applied to physiological signals [38], [40]. Among Katz’s FD, Petrosain’s FD, and Higuchi’s FD, the latter one provides the most accurate estimates of the FD for synthetic and biomedical data [38].

Entropy is a powerful and broadly-used nonlinear metric used to assess the dynamical characteristics of time series [41], [42]. Shannon entropy and conditional entropy respectively show the amount of information and the rate of information production [43]–[45]. Based on these concepts, sample entropy (SampEn) [42], dispersion entropy (DispEn) [45], fluctuation DispEn (FDispEn) [46], and local fuzzy entropy (LocFuzEn) [47] are utilized in this study. SampEn, which is based on conditional entropy, shows the negative natural logarithm of the conditional probability that two series similar for *m* sample points remain similar at the next sample, where self-matches are not considered in calculating the probability [42]. LocFuzEn, as a modification of SampEn, uses a fuzzy membership function instead of the the Heaviside function used in the SampEn algorithm. DispEn is based on symbol entropy and Shannon entropy considering the probability of each dispersion pattern in a signal [45]. FDispEn, which is based on Shannon entropy and fluctuation-based dispersion patterns, estimates the dynamical variability of the fluctuations of signals [46].

### F. COMPLEXITY

Entropy methods may fail to account for the multiple time scales inherent in biomedical recordings [44], [48]. Thus, we use multiscale methods [49] to quantify complexity or the physiological dynamics over multiple time scales. The complexity in signals denotes “meaningful structural richness” [44]. The complexity-based algorithms include two main steps: (1) coarse-graining process; and (2) calculation of SampEn, DispEn, FuzEn, or FDispEn at each temporal scale *τ* [44], [50]. The coarse-grianing process at scale factor *τ* can be considered as a low-pass filter with cutoff frequency $\frac{fs}{2\tau }$. In this study, we use multiscale DispEn (MDE) [51], multiscale FuzEn (MFE) [48], and multiscale FDispEn (MFDE) [49].

### G. FUNCTIONAL CONNECTIVITY

Connectivity measures seek to quantify the relationship between two time series or sensors [52]. To detect changes in connectivity between the right and left iris centers, we use four main connectivity approaches, including linear model-free correlation, time-domain nonlinear correlation, frequency-domain linear coherence, and time-domain linear model-based Granger causality in the frequency bands 0.08–5 Hz and 5–25 Hz [52], [53].

### H. STATISTICAL ANALYSIS

In this study, the non-parametric Mann-Whitney *U* test is employed to evaluate the differences or effect between two sample means that come from the same population, and used to evaluate if two sample means are equal or not. The Hedges’ *g* effect size [54] is also used to quantify the differences between the results for two groups [55]. The Hedges’ g test shows the difference between the means of two groups, divided by the weighted average of standard deviations for these two groups.

## III. RESULTS AND DISCUSSION

### A. MATCHED FILTERING

As shown in Figure 4, saccades recorded from ataxia subjects without saccadic dysmetria were significantly more similar to the ideal template compared with individuals with dysmetria (*p <* 1*e –* 10, Mann-Whitney *U* test). Additionally, the Hedges’ *g* effect size (es) for the results for the individuals with vs. without dysmetric saccades shows that the difference between the two groups was large (es = 1.18).

### B. THRESHOLD-BASED SURFACE

**FIGURE 6.**
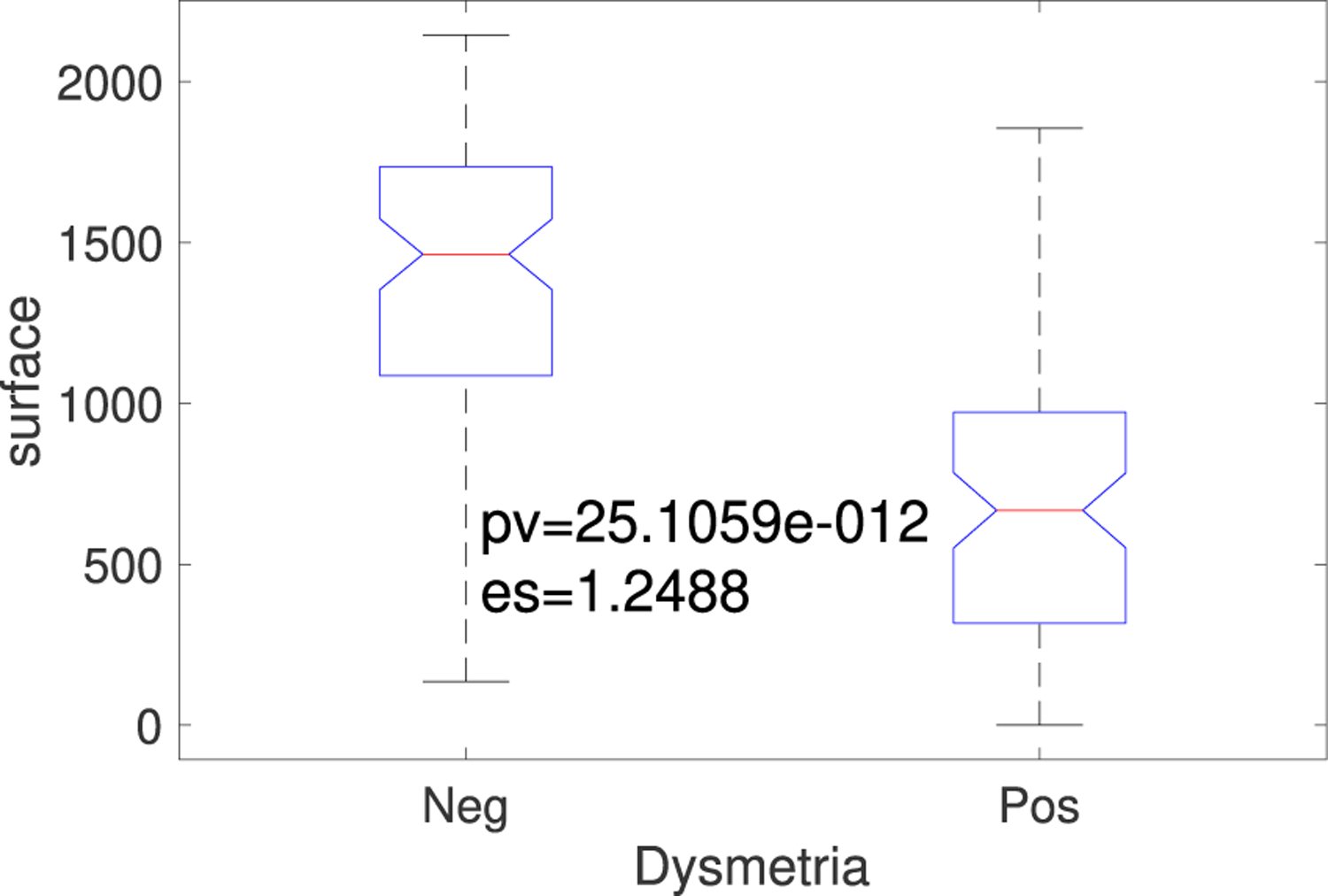
Boxplot based on the area above the positive threshold and the signal plus the area below the negative threshold and the signal for the individuals with vs. without dysmetria (see Figure 6), and the *p*-value and effect size for these two groups.

The area above the positive threshold and the signal plus the area below the negative threshold and the signal for the patients with vs. without dysmetria are shown in Figure 6. The *p*-value and effect size demonstrate that this approach significantly and strongly discriminated the two groups. The positive and negative thresholds were set to 0.35 and −0.35, respectively. Other thresholds (+/−0.4 to +/−0.2) led to similar findings.

### C. ENTROPY, FRACTAL DIMENSION, AND LEMPEL-ZIV COMPLEXITY

The values for Higuchi’s FD, LZC, DispEn, FDispEn, SampEn, LocFuzEn in the frequency band 0.08–5 Hz for individuals with and without oculomotor dysmetria are shown in Table 1. Individuals with oculomotor dysmetria had higher values indicating that the eye tracking signal was more irregular/uncertain than those without dysmetria in the frequency band 0.08–5 Hz. The effect sizes and *p*-values are also presented in Table 1. Based on the effect sizes, DispEn was the best algorithm in discriminating the two groups in the frequency band 0.08–5 Hz. There was no significant difference between the two groups in the frequency band 5–25 Hz. This observation suggests the importance of lower frequency components of saccade data in detecting dysmetria.

### D. COMPLEXITY

**FIGURE 7.**
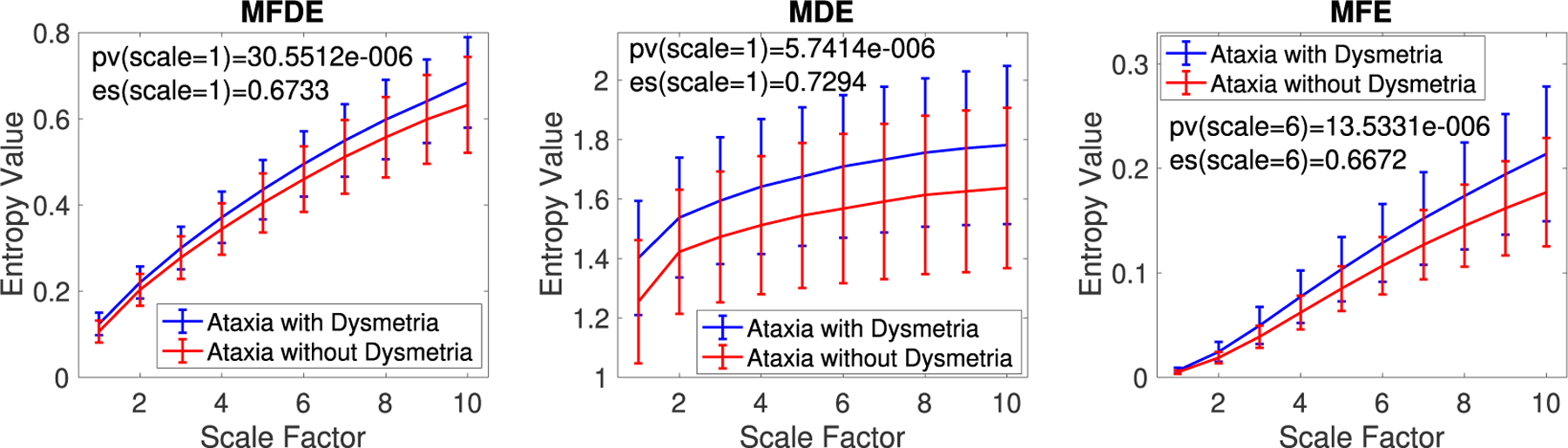
Mean and SD of the MFDE, MDE, and MFE values for the individuals with and without dysmteria and their maximum *p*-values and effect sizes for the discrimination of the two groups.

The MFDE, MDE, and MFE results for the frequency band 0.08–25 Hz, shown in Figure 7, demonstrate that eye tracking data from individuals with dysmetria are more complex than those without dysmetria. The results show that for all the frequency bands from 0.08–25 Hz (scale factor 1) to 0.08–12 Hz (scale factor 10), the saccadic dysmetria data are more irregular or uncertain than saccades without dysmetria. The effect sizes and *p*-values, shown in Table 7, indicate that MDE was the best algorithm in distinguishing individuals with and without dysmetria.

### E. FUNCTIONAL CONNECTIVITY

We used the Connectivity techniques to test the hypothesis that movement of the left and right eyes is less synchronized in individuals with oculomotor dysmetria compared to individuals without dysmetria. The functional connectivity indexes using coherence, nonlinear correlation, Granger causality, and correlation in the frequency bands 0.08 to 5 Hz and their corresponding *p*-values and effect sizes are demonstrated in Table 1. The right and left iris centers for patients without dysmetric saccades were more synchronized, as assessed by nonlinear and linear correlation and Granger causality. Note that there is no significant difference between these two groups in the frequency band 5–25 Hz.

## IV. CLASSIFICATION

Next, we trained a classification model to distinguish individuals with dysmetric saccades from individuals without dysmetric saccades. Features from the best technique in each category of methods employed above (i.e., template matching, threshold, MDE, DispEn, and nonlinear correlation) were combined as input to the model. Given the relatively large number of features (14), we first reduced the dimensionality of the input using principal components analysis (PCA) [56].Based on the cumulative explained variance ratio as a function of the number of components, we chose 5 components that capture at least 95% of the variance. A linear support vector machine (SVM - using 10-fold cross validation) was then used to train and test the ability of the first five principal components of the feature space to distinguish 72 ataxia subjects with dysmetria from 80 ataxia and Parkinson’s individuals without dysmetria. The model achieved 78% accuracy (sensitivity 80% and specificity 76%). This is worth mentioning that we also used a kernel PCA and Radial Basis Function (RBF) kernel SVM. However, there was no noticeable between the results obtained by the kernel PCA and RBF kernel SVM and PCA and linear SVM. Thus, we have decided to use the simpler approach (PCA + linear SVM).

## V. TEST-RETEST RELIABILITY

To evaluate the test-retest reliability of the the best technique in each category of methods employed above for the detection of oculomotor dysmetria, we used Pearson correlation coefficient (PCC) for 12 subjects with two recording sessions. The average (standard deviation) duration between the two sessions across those 12 individuals with repeats is equal to 10.6 ± 3.5) months. The PCC values, presented in Table 2, show that the template matching, threshold, and nonlinear correlation techniques lead to acceptable reliability.

## VI. DISCUSSION AND CONCLUSION

In this study, we demonstrated the ability of computer vision and signal processing methods applied to mobile device video data to capture dysmetric saccades, which is a key eye movement abnormality in cerebellar ataxias. We showed that applying a range of signal processing methods to the short eye tracking data enabled us to distinguish between individuals with normal and abnormal saccades with high accuracy. To our knowledge, detection of dysmetric saccades from a consumer-grade mobile device has not been previously reported although there is a trend in using inexpensive, easy to use eye trackers, which are noticeably more affordable than the commercial grade systems [57]–[59].

We used various signal processing methods based on template matching, information theory (complexity- and irregularity-based techniques), and functional connectivity to distinguish individuals with and without saccade abnormalities. Among them, the lowest *p*-values and largest effect sizes were obtained using the matched filtering and threshold-based surface methods in the frequency band 0.08 to 5 Hz. This result suggests the importance of this frequency band in detecting saccadic dysmetria for ataxia patients. In the frequency band 0.08–5 Hz, SampEn, DispEn, FDispEn, MDE, MFDE, and MFE significantly distinguished individuals with and without dysmetria with moderate or large effect sizes. The right and left iris centers for patients without dysmetric saccades were more synchronized compared using nonlinear correlation, Granger causality, and correlation.

There are now a number of disease modifying drug development programs aimed at slowing or stopping the progression of spinocerebellar ataxias [60]–[62]. As these and other therapies for cerebellar ataxias come to the market it will become even more important to have widely available and inexpensive screening tools for early detection of disease to enable treatment early in the disease course. The mobile phone-based approach for saccade assessments developed here could be an important component of a scalable screening tool for ataxias, in addition to speech and motor control components [63]–[66].

Drug development efforts are in need of improved clinical outcome measures that are sensitive to disease progression and capture meaningful behaviors. Improved outcome measures could enable a more efficient evaluation of potential therapies by reducing the size, duration, and cost of clinical trials. Oculomotor assessments are relatively unexplored as a potential clinical outcome measure in ataxias, in part because of the requirements for specialized and expensive equipment, dedicated space, and training. In future work, the approach for saccade assessments here could be relatively easily incorporated into natural history studies and clinical trials to evaluate changes in saccade features with disease progression. Furthermore, the signal processing techniques used to quantify saccades could be applied to oculomotor data generated from other eye tracking technologies. Outcome measures for clinical trials generally require that the outcome is clinically meaningful. While saccades play an important role in human behavior and function [16], to our knowledge it is an open question as to if and how dysmetric saccades affect functions such as reading, multitasking, and other information processing tasks. Addressing this question is a goal for future work.

This is worth mentioning that the threshold-based surface approach is only able to detect overshoots of the intended position with eyes and so, this is not good enough for the detection of both the overshoots and undershoots seen in dysmetria. However, entropy, fractal dimension, complexity, functional connectivity, and template matching methods are able to detect both the overshoots and undershoots of eye movement. Since we use all these methods for our classification, our approach can detect both the overshoots and undershoots.

In spite of the promising findings and high classification accuracy, other existing and novel signal processing and machine learning methods can be developed in future studies. For example, time-frequency methods, such as variational mode decomposition and empirical mode decomposition, can be employed in future studies to characterize the spectral content of dysmetric saccades. Additionally, new techniques can be utilized for improved blink removal. Herein, we assessed the methods in the frequency bands 0.08–5 Hz and 5–25 Hz. However, in future work, these same signal processing techniques can be used to evaluate the effect of dysmetria on smaller frequency components that lie outside of the studied range. Finally, the physiological nature of the findings for functional connectivity and entropy methods for saccadic dysmetria in different frequency bands needs to be further investigated.

## Figures and Tables

**TABLE 1.** Mean and standard deviation of the Higuchi’s FD, LZC, DispEn, FDispEn, SampEn, and LocFuzEn values as well as coherence, nonlinear correlation, Granger causality, and correlation coefficients for the individuals with and without dysmetria in the frequency band 0.08–5 Hz, and their corresponding *p*-values and effect sizes.

Method	Positive dysmetria	Negative dysmetria	*p*-value	Effect size
Higuchi’s FD	1.09 ± 0.01	1.08 ± 0.01	0.55	0.05
LZC	0.09 ± 0.01	0.09 ± 0.01	0.75	0.05
DispEn	1.41 ± 0.19	1.28 ± 0.21	68.18e-6	0.64
FDispEn	0.12 ± 0.02	0.10 ± 0.02	67.26e-6	0.62
SampEn	0.03 ± 0.01	0.02 ± 0.01	31.32e-6	0.59
LocFuzEn	0.09 ± 0.04	0.08 ± 0.03	0.09	0.31

Coherence	0.89 ± 0.09	0.90 ± 0.06	0.76	0.14
Nonlinear Correlation	0.97 ± 0.02	0.98 ± 0.01	0.01	0.41
Granger Causality	0.05 ± 0.03	0.07 ± 0.03	0.01	0.36
Correlation	0.96 ± 0.08	0.98 ± 0.02	0.01	0.28

**TABLE 2.** Pearson correlation coefficient (PCC) for 12 subjects with two recording sessions using template matching, threshold, MDE (scale 1), DispEn, and nonlinear correlation.

template matching	threshold	MDE (scale 1)	DispEn	nonlinear correlation
0.78	0.67	0.50	0.55	0.8366
